# What is the significance of guidelines in the primary care setting? Results of an exploratory online survey of general practitioners in Germany

**DOI:** 10.1007/s10354-021-00849-3

**Published:** 2021-06-08

**Authors:** Julian Wangler, Michael Jansky

**Affiliations:** Centre for General Medicine and Geriatrics, Mainz University Medical Centre, Universitätsmedizin Mainz, Am Pulverturm 13, 55131 Mainz, Germany

**Keywords:** Clinical guidelines, Implementation, Adherence, Attitudes, General practitioner, Klinische Leitlinien, Implementierung, Adhärenz, Einstellungen, Hausarzt

## Abstract

Medical guidelines aim to ensure that care processes take place in an evidence-based and structured manner. They are especially relevant in outpatient primary care due to the wide range of symptoms and clinical pictures. In German-speaking countries, there is a lack of current findings documenting general practitioners’ opinions and experiences regarding guidelines, their expectations and their views on what improvements could be made to increase the use of this type of evidence-based instrument in the primary care setting. Between April and August 2020, a total of 3098 general practitioners were surveyed in the states of Baden–Württemberg, Hesse and Rhineland–Palatinate via an online questionnaire. Alongside the descriptive evaluation, t‑testing was used to determine significant differences between two independent sampling groups. A factor analysis was also used to cluster the expectations of those surveyed regarding the fulfilment of requirements relating to guidelines. A total of 52% of those surveyed have a positive view of guidelines. Overall, guidelines are associated with an increased evidence-based approach (69%), standardisation of diagnosis and treatment (62%) and a reduction in overprovision or underprovision of care (57%). In all, 62% of the physicians who implemented guidelines observed positive effects on the quality of care provided, and 67% reported that the implementation of guidelines improved the quality of their diagnostic or therapeutic skills. However, implementation is often seen as being complicated (43%) and restricting the physician’s ability to act independently (63%). Survey participants suggested that guidelines could be optimised by giving greater consideration to nondrug alternatives (46%), focusing on issues related to quality of life (42%) and offering a comparative assessment of various treatment options (39%). In order to further promote the attractiveness of guidelines for primary care the design of guidelines should be oriented more towards their application; they should be well-presented to make them easier to implement. The scope of action available to the physician should be stressed. The guidelines should provide recommendations on opportunities for the delegation of tasks within practice teams.

**Supplementary Information:**

The online version of this article (10.1007/s10354-021-00849-3) contains supplementary material, which is available to authorized users.

## Abstract (de)

Medizinische Leitlinien sollen einen Beitrag dazu leisten, dass Versorgungsprozesse evidenzbasiert und strukturiert ablaufen. Insbesondere für die ambulante Primärversorgung, die mit einer Vielzahl von Symptomatiken und Krankheitsbildern konfrontiert ist, sind sie relevant. Für den deutschsprachigen Raum mangelt es an aktuellen Befunden, welche Einstellungen und Erfahrungen Hausärzt*innen in Bezug auf Leitlinien vertreten, welche Erwartungen sie haben und welche Verbesserungsmaßnahmen ihrer Ansicht nach ergriffen werden sollten, damit die hausärztliche Bereitschaft zur Nutzung solcher evidenzbasierter Instrumente weiter steigt. Mittels einer Online-Befragung wurden zwischen April und August 2020 insgesamt 3098 Hausärzt*innen in den Bundesländern Baden-Württemberg, Hessen und Rheinland-Pfalz befragt. Neben der deskriptiven Auswertung kam zur Feststellung von signifikanten Unterschieden zwischen 2 Gruppen ein t‑Test für unabhängige Stichproben zum Einsatz. Zudem wurde eine Faktorenanalyse durchgeführt, um Erwartungen der Befragten in Bezug auf die von Leitlinien zu erfüllenden Voraussetzungen unterschiedlichen Clustern zuzuordnen. In Bezug auf Leitlinien sind 52 % der Befragten positiv eingestellt. Leitlinien werden allgemein mit verstärkter Evidenzorientierung (69 %), einer Vereinheitlichung von Diagnose- und Behandlungsstandards (62 %) und einem Abbau von Über- oder Unterversorgung (57 %) verbunden. Insgesamt 62 % der Ärzte, die Leitlinien einsetzen, beobachteten positive Effekte für die Versorgungsqualität, und 67 % bekundeten, durch die Anwendung von Leitlinien die eigenen diagnostischen bzw. therapeutischen Kompetenzen verbessert zu haben. Indes wird die Implementierung oft als kompliziert (43 %) und als Einschränkung der ärztlichen Handlungsfreiheit (63 %) erlebt. Zur weiteren Optimierung wird v. a. eine stärkere Berücksichtigung nichtmedikamentöser Alternativen (46 %), eine Thematisierung von Fragen der Lebensqualität (42 %) und ein Vergleich von Therapieoptionen (39 %) angeregt. Um die Attraktivität von Leitlinien für die Hausarztmedizin weiter zu fördern, sollten Leitlinien anwendungsnah und übersichtlich gestaltet sein. Auch sollten ärztliche Handlungsspielräume betont werden. Die in Leitlinien gegebenen Empfehlungen sollten ferner Möglichkeiten der Delegation innerhalb des Praxisteams aufzeigen.

## Introduction

Clinical guidelines are considered as an important instrument for effective, practical and evidence-based care provision [1–3]. Published by major scientific medical societies, they can be seen as an institutionally compiled framework. The objective is to support physicians in their decisions on appropriate diagnostics or treatment in specific disease-related situations [4–6]. The guidelines of the scientific medical societies can therefore be described as systematically developed aids for medical decision-making that provide concrete recommendations for action. They are based on current scientific knowledge and methods that have been tried and tested in practice and aim to ensure more safety in patient care, but also take economic aspects into account. There are usually different levels of evidence that indicate how well-founded and proven a guideline is [4]. Regular reviews and updates are carried out by the guideline commissions of the respective specialist societies. In most cases, clinical guidelines are not legally binding for physicians and therefore neither establish nor discharge liability.

In German-speaking countries, the development of clinical guidelines for outpatient care have gained momentum, especially from the late 1990s. There is great potential for the application of guidelines in the primary care setting. In particular, as outpatient primary care providers, general practitioners face an enormous range of symptoms and clinical pictures [7]. Therefore, there is an obvious need for systematic decision-making tools to aid structures for diagnostic clarification, disease monitoring and disease management [8, 9]. Guidelines can also help general practitioners (GPs) to quickly implement the necessary steps for the appropriate (further) treatment of patients, for example, in cases where cooperation with specialists or other specific supportive healthcare services is required [10]. Despite such advantages, clinical guidelines are also a subject of criticism, especially in outpatient care. For example, the objection is made that guidelines restrict the freedom of treatment for doctors and, due to their strict specifications, act as a limiting factor in patient care. In addition, guidelines are often associated with the increasing economization of the health system [7].

The focus of this article is on general practitioner care in Germany, for which guidelines are mainly provided by the German College of General Practitioners and Family Physicians (DEGAM). Over the last two decades, the DEGAM has developed more than two dozen evidence-based primary care setting guidelines [11]. Particularly noteworthy is that active general practitioners are involved in various development stages and testing processes to ensure the highest possible applicability levels [12].

This type of involvement is advantageous to ensure that guidelines achieve their full potential in everyday care. For this, they require a willingness from the physician to implement the recommendations in the practical setting. Therefore, this depends a great deal on physicians recognising the specific benefits of implementing guidelines and adhering to them [3, 13].

International studies have shown that general practitioners determine the value and usefulness of clinical guidelines from the perspective of diagnostic clarification [14]. However, this group of physicians more frequently voices criticism on guidelines. GPs are critical of guidelines due to the restrictions they place on the physician’s scope of action, their disproportionate effects on established practice routines (excessive strain on time and resources) and the ongoing economisation in the healthcare sector as a result of guidelines [15–20]. A further aspect resulting in a more sceptical stance from general practitioners are drug recommendations in quantitative and qualitative studies from Europe, Canada and the United States [19, 21, 22]. Many of the physicians surveyed believe that these recommendations are often excessive and result in significant side effects, for example, the drug-based treatment of dementia patients. There is also concern that evidence-based guidelines could lead to an excessive burden being placed on physicians since they are frequently updated and, in some cases, recommend complex diagnostic and therapeutic approaches [23]. GPs are notably more uncertain about the legal status, accessibility and evidence of guidelines than other doctors [15]. In addition, part of the primary care doctors feel pressured to use guidelines on a regular basis [5, 13, 15, 18]. Some GPs also criticize that insurance companies tend to blame physicians if they are not regularly using guidelines.

In German-speaking countries, there is a current lack of findings documenting the range of opinions and experiences of general practitioners regarding guidelines and to what extent they follow them [1, 24]. More research is required on the conditions under which GPs envisage implementing guidelines [1].

As with the aforementioned international studies, there is older research available into general practitioners in Germany that provides insight into why they have a more reserved stance toward guidelines while rarely possessing in-depth knowledge on the subject [25]. The reasons listed are that guidelines are often seen as interfering with therapy freedom [1, 3, 24]. It is also shown that general practitioners find that externally collected evidence occasionally contrasts with what they experience in the anamnestic process during direct patient consultation (experience-based knowledge, in-depth understanding of treatments) [13, 26–28]. Vollmar et al. determined that, despite all efforts focused on the broad implementation of DEGAM guidelines, GPs often claim they are either “not aware” of them or that they are “rarely used” [1, 3].

### Research interest

An explorative online questionnaire was used to obtain current information on this practice-relevant topic. The focus of the study is on GP-based guidelines in Germany. The research interest can be summarised in the following main questions:

- What is the opinion of GPs with regard to guidelines?
- What expectations do they have of guidelines?
- In which fields of application are guidelines used? What experiences have GPs had with them?
- How well known are DEGAM guidelines and their benefits?
- What must be improved to make the guidelines more attractive for GPs in the future?
- What are the differences between GPs who regularly implement guidelines and those who avoid them?

## Materials and methods

### Study design and setting

The present study aimed to obtain as broad an image as possible of general practitioners’ opinions and experiences regarding guidelines. The choice was made to use a quantitative online survey of GPs with an invitation letter sent by post. The study was carried out between 15 April and 15 August 2020 in Baden–Württemberg, Hesse and Rhineland–Palatinate. It was conducted and processed by the authors, two primary care researchers employed at a scientific department of general medicine.

### Questionnaire and sociodemographic variables

Various elements were incorporated into the questionnaire development process (see supplementary material):

- The results of a previous group discussion with ten GPs on the topic of guideline orientation
- The use of relevant previous studies by the authors as a foundation [5, 29]
- Literature research, which led to various studies being incorporated into the development process (especially [1, 24])

The content blocks covered by the questionnaire correspond to the listed research questions. The sociodemographic characteristics of age, gender, practice environment, type of practice, and patients per quarter were recorded. Before beginning the field study, a pretest with 25 general practitioners was carried out. This was a convenience sample in which the questionnaire was presented to some of the department’s lecturers, who are all general practitioners. This pretest was primarily about comprehensibility and completeness. Following the pretest, several items were slightly modified (question 3, 4, 8).

### Recruitment and participants

A total of 13,170 active general practitioners inBaden–Württemberg (6664), Hesse (3839) andRhineland–Palatinate (2667) were invited to participate in the anonymised questionnaire via a postal invitation. The cover letter was sent only once and contained the key to access the password-protected online questionnaire set up on the department’s portal using LimeSurvey for Windows.

Written informed consent for participation was obtained from all participants before the start of the online questionnaire. The participants received no compensation or incentives.

### Ethics

During this study, no sensitive patient data was gathered or clinical tests performed. This is a strictly anonymised survey of a total of 3098 general practitioners. However, the authors contacted the Ethics Commission of the State of Rhineland–Palatinate, Germany, before beginning the study to ensure that it conformed with the medical professional code of conduct. The Ethics Commission informed us that approval by an ethics committee was not necessary.

### Data analysis

Due to the online survey via LimeSurvey, the data could be transferred directly to the statistical analysis. We analysed the data using SPSS 23.0 for Windows. Apart from the descriptive analysis, we used a *t*-test for independent samples to analyse for any significant differences between the two groups, assuming significance at values of *p* ≤ 0.001. Some of the Likert-scale gradations were summarised to illustrate the results.

In order to better display the different expectations (questionnaire question 15) of GPs regarding guidelines, the method of factor analysis (Varimax rotation) was used, in which variables are combined into factors on the basis of systematic relationships (correlations) [30]. The conditions for factor analysis were assessed beforehand (sample suitability according to Kaiser–Meyer–Olkin, significant Barlett test results for the sphericity and commonality of all incorporated variables over the threshold value of 0.5). The chosen threshold value for an item to be factor loaded was 0.4/−0.4 [30].

## Results

### Sampling

Of the 3167 questionnaires processed, a total of 3098 fully completed questionnaires were included in the evaluation (response rate: 24%). The sampling is structured as follows:

- Gender: 51% male, 49% female
- Average age: 54 (median: 55)
- Practice environment: 49% mid-sized and large cities, 51% countryside and small towns
- Practice type: 55% single-partner practices, 45% joint practices
- Patients per quarter: 20% < 1000, 20% 1000–1500, 32% 1501–2000, 28% > 2000

### Opinions on guidelines

In all, 52% of those surveyed have a positive or very positive view of guidelines; 40% expressed a negative opinion (8% responded with “difficult to say”). Based on opinion or experience, 48% find guidelines to be very useful or somewhat useful, in contrast to the 41% who find them to be somewhat less or not very useful (7% see no benefit, 4% no response). While 73% of physicians located in mid-sized and large cities had a (very) positive opinion of guidelines, this opinion was shared by only 33% of physicians based in small cities and the countryside (*p* < 0.001). In comparison, 74% of urban physicians see guidelines as (very) useful, with only 24% of countryside physicians sharing this view (*p* < 0.001).

Furthermore, 69% find guidelines help boost a more evidence-based approach and the application of current knowledge; 62% agree with guidelines to standardise diagnosis and treatment; 57% view guidelines as useful in helping to reduce the overprovision, underprovision, and incorrect provision of care; and 42% perceive improved cooperation between the various care providers (e.g., general practitioners and specialists).

Those surveyed associated guidelines with application potentials such as improved structuring and increased efficiency of diagnoses or therapies (Table 1). In contrast, the integration of guidelines into practice procedures is not always deemed to go smoothly. The majority see guidelines as restricting physicians’ scope of action. In this aspect, there is a notable difference between urban physicians (36%) and those based in the countryside (88%, *p* < 0.001), as well as between physicians below and above the average age (42% to 83%, *p* < 0.001).

**Table 1:** Opinion-related statements on guidelines. Question:* Which of the following statements do you agree with?* (*N* = 3098; the response *categories Fully agree/Somewhat agree* and *Somewhat disagree/Fully disagree* were combined)

	Fully agree/Somewhat agree (%)	Somewhat disagree/Fully disagree (%)
“Guidelines enable a more structured approach to diagnosis and therapy.”	88	12
“I prefer to rely on my own approach rather than guidelines.”	77	33
“There is often not enough time in the everyday practice setting to implement guidelines.”	63	37
“Guidelines place too many restrictions on therapy freedom.”	63	37
“Guidelines interfere too much in established practice procedures and routines.”	62	38
“Guidelines increase the efficiency of diagnostic procedures and therapies in the medical practice setting.”	58	42
“The recommendations for action provided in guidelines often coincide with my personal experience as a physician.”	57	43
“Guidelines are generally easy and straightforward to implement.”	40	60

### Use of guidelines

A total of 27% report that they often use guidelines, while 27% use them occasionally and 35% use them rarely (11% never). Guidelines are mainly used in case of suspicion (55% often or occasionally), during initial diagnosis (60% often or occasionally), during therapy or disease management (54% often or occasionally), and during check-ups (48% often or occasionally).

Of the physicians that implement guidelines often, occasionally, or rarely (*n* = 2725), 62% report that the guidelines they implemented had an overall very positive or somewhat positive effect on care and treatment quality (25% somewhat negative, 13% no response). There are notable differences between urban (72% positive) and rural practice locations (43% positive, *p* < 0.001).

In addition, 67% report that the implementation of guidelines led to an improvement in their diagnostic or therapeutic skills, 59% prefer to use guideline recommendations for drug therapies and 52% consider the use of guidelines to be essential for the provision of care. Guidelines bring more advantages than disadvantages for 65% of those surveyed.

Despite these predominately positive experiences, 43% of those surveyed reported often or occasionally experiencing restrictions or complications in the practice setting as a result of implementing guidelines. Physicians with a more sceptical stance toward guidelines report this same issue more than three times more frequently (80%) than those with a positive stance (24%, *p* < 0.001).

### Awareness of guidelines for general practitioners

The majority of those surveyed are aware of the guidelines listed below published by the German College of General Practitioners and Family Physicians (DEGAM), which have been implemented by some of these general practitioners:

- Stroke (88% aware; 63% implemented)
- Fatigue (83% aware; 35% implemented)
- Chest pain (82% aware; 59% implemented)
- Multi-medication (77% aware; 60% implemented)
- Cardiovascular prevention (64% aware; 32% implemented)
- Sore throat (63% aware; 56% implemented)
- Acute and chronic coughing (62% aware; 25% implemented)
- Acute vertigo (59% aware; 33% implemented)
- Multimorbidity (54% aware; 37% implemented)

For two-thirds of those surveyed, the fact that the guidelines are issued by DEGAM is important. Under this condition, two-thirds (68%) reported that they prefer to implement these guidelines over guidelines from other professional associations.

### Expectations of guidelines and optimisation approaches

Those surveyed formulate various requirements for the nature of guidelines so that they consider using them (Table 2). Alongside the exclusion of liability risks, particular emphasis is placed on user-friendliness. This includes a complexity-reducing algorithm that acts as a decision-making benchmark during diagnostic and therapeutic tasks. An essential requirement for many of those surveyed is the involvement of general practitioners in the guideline development process.

**Table 2:** Guideline requirements. Question: *In your opinion, what must a* *good set of guidelines have for you to consider implementing it? Please indicate how important the following aspects are to you. *(*N* = 3098; response categories *Very important/Somewhat important* were combined)

		Rotated Component Matrix			
	Very important/ Somewhat important (%)	Component 1(Variance Explanation: 56.8%)	Component 2(Variance Explanation: 13.8%)	Component 3 (Variance Explanation: l.: 10.6%)	Component 4 (Variance Explanation: 9.5%)
It should be as easy as possible to implement	90	0.401	0.804	0.277	−0.047
It must be ensured that the recommendations have a sound legal basis	87	0.304	0.904	0.074	0.131
It should provide easily comprehensible algorithms or diagnostic and therapeutic approaches (i.e., using diagrams)	85	0.884	0.302	0.200	−0.086
It must specify red flags, i.e., particularly important warning signs that indicate clinical pictures in need of further clarification	84	0.768	0.531	0.194	−0.030
The benefits of its recommendations for action must be evidence-based and scientifically valid	83	0.909	0.213	0.221	0.074
General practitioners should be involved in the development of guidelines or have tested guidelines in a practice setting before publication	79	0.028	0.447	0.833	0.168
The recommendations for action should conform to the applicable fee schedule to ensure that physician costs are covered	76	0.134	0.882	0.072	0.324
It should provide concrete ranges for laboratory values (e.g., for blood testing)	65	0.757	0.367	0.043	0.454
It should provide clear information on when or for how long a wait-and-see approach is appropriate and when referral is indicated	64	0.752	0.323	0.117	0.476
The guidelines must provide intelligent recommendations for the delegation of tasks for the entire practice team	51	0.292	−0.037	0.880	0.117
It should be an S3 guideline (highest evidence level)	41	−0.016	−0.111	0.736	0.554
Guideline-compliant training courses must be available	39	0.091	0.230	0.240	0.892

Extraction Method: Principal Component Analysis; Rotation Method.: Varimax, Kaiser Normalization; Rotations converge in 8 iterations; Total Variance Explained: 90.7%; Kaiser–Meyer–Olkin Sampling Adequacy: 0.69; Bartlett Significance Level: *p* < 0.001

As shown in the factor analysis, the expectations of those surveyed relating to requirements that guidelines must fulfil can be classified into four large clusters of different sizes. The first two clusters are predominately characterised by the topics of applicability and practicality for GPs; the remaining clusters highlight specific guideline characteristics (e.g., task delegation recommendations, training format compatibility). A further condition for the implementation of guidelines is a high quality and evidence standard (S3 type).

Greater consideration of non-drug alternatives is primarily desired (46%) to optimise guidelines for general practitioners, followed by an increased focus on quality-of-life issues (42%), comparative evaluation of different therapeutic options (39%), and the inclusion of alternative medicine (35%). In addition, 25% of those surveyed would like more information on the effectiveness of recommended therapies. Furthermore, 49% want the guidelines to have a more compact and concise design.

If the desired changes are implemented, 7% can imagine increased guideline use in the future, while 76% are considering some form of an increase in implementation (17% reported no change in their current stance).

### Differences between guideline users and nonusers

Differences can be ascertained between respondents who often or occasionally use guidelines (*n* = 1638) and those who rarely or never use them (*n* = 1460). Of regular users, 38% believe that guidelines restrict therapy freedom too much, while 89% of physicians who avoid them share this opinion (*p* < 0.001). Of the frequent or occasional users, 91% report that guidelines generally correspond to their own experiences, while only 19% of infrequent users or nonusers report the same (*p* < 0.001).

In all, 47% of nonusers would like general practitioners to be more closely involved in the development process, as would 9% of regular users (*p* < 0.001). Furthermore, 62% of those who do not use guidelines would prefer stronger consideration to be given to non-drug alternatives during guideline development, whereas among frequent and occasional users this figure is 27% (*p* < 0.001). While 47% of nonusers stated that there should be increased inclusion of alternative medicine, 22% of regular users share this opinion (*p* < 0.001). In addition, 75% of infrequent or nonusers can imagine using guidelines more frequently in the future should their preferred optimisation recommendations be implemented.

## Discussion

### Main findings and comparison with prior work

The survey of 3098 general practitioners illustrated that the majority consider guidelines to be an important decision-making instrument which can provide evidence-based, structured and effective care. Where guidelines are used, the assessment is even more positive. The majority of respondents stated that the implementation of guidelines has improved their proficiency and that now they would not wish to forgo their use. For those surveyed, it is particularly important for guidelines to be easy to implement and legally sound. In addition, an intuitive and concise design and adherence to the applicable fee schedule were highlighted. However, there is also criticism, with guidelines often being described as being poorly compatible with established practice processes. A further concern is that they may restrict therapy freedom.

The results of the present study correspond to national and international research findings that identified a critical and distant underlying attitude of general practitioners toward guidelines [3, 12–22, 25–28]. GPs see the potential for the application of guidelines in order to quickly implement the necessary steps for the appropriate (further) treatment of patients [7–10]. Despite such advantages, they fear that guidelines may restrict their flexibility and freedom of choice in a crucial way [7].

However, in comparison to prior studies, the results of the survey reflect a growing acceptance, knowledge, and implementation of general practitioner guidelines. In this respect, the results correspond to the positive findings of a recent study of general practitioner opinions relating to disease management programmes [29]. Today, the work of many GPs is increasingly based on the use of standardised evidence-based interventions [12, 31].

In the course of the evaluation it became apparent that urban physicians had a notably more positive attitude towards clinical guidelines than rural physicians [20]. Also, younger physicians have a far more favourable opinion of guidelines than older physicians. These findings correspond to other studies [26, 27]. A possible explanation for these findings could be that doctors in urban environments are more used to structured care processes due to a complex local care system. Younger doctors (more likely to be found in urban regions) are used to the fact that guidelines are available for a wide variety of clinical pictures and have already come into contact with such instruments in the course of their medical studies.

The survey also revealed considerable differences between regular guideline users and those who do not use guidelines [24]. The latter group would like to see increased general practitioner involvement in guideline development and more consideration of non-drug alternatives and alternative medicine.

DEGAM guidelines for cardiovascular issues, fatigue, and multi-medication are widely known and used by those surveyed. Physician willingness to implement guidelines depends significantly on the guidelines having been issued by DEGAM with the involvement of general practitioners [1, 12, 24].

### Strengths and limitations

The survey was underpinned by a previous discussion and achieved an adequate response rate. It does, however, exhibit a range of limitations that must be critically examined. The study cannot claim to be representative due, on the one hand, to the limited number of cases and the regional recruitment, and on the other hand, to the fact that it was an online survey. It can be assumed that this form of data collection was not equally suitable for all participating GPs, for example, due to a lack of online affinity or insufficient internet connection in more rural locations.

In addition, it cannot be ruled out that physicians with a thematic interest or positive previous experience with guidelines participated to a greater extent, so a selection bias could be present.

Furthermore, it should be noted that the study focused on the collection of (self)assessments relating to the use of guidelines, which do not necessarily fully correspond with actual behaviour. Due to the complexity of the topic, the questionnaire can document only a small segment of guideline-related opinions. Therefore, the study should be viewed as an explorative article that does not replace assessment and evaluation of the use of specific guidelines.

The study dealt mainly with GP-based guidelines in Germany. Therefore, the results of the general practitioner survey cannot simply be transferred to other countries. Nevertheless, the authors assume that they were able to capture basic attitudes and perspectives of primary care providers.

## Conclusion

It was demonstrated that many general practitioners see that implementing guidelines offers significant benefits to patient care. However, this positive perception and willingness to use guidelines is more prevalent among urban and younger physicians. There is still a significant proportion of physicians who continue to have a reserved stance toward guidelines and rarely use them.

The question of the distribution and implementation of primary care guidelines is based on complex research, evaluation, planning, and implementation processes. A specific approach is required focusing on the various target groups (early adopters vs laggards, teams with task delegation structures, etc.). To promote the implementation of guidelines among general practitioners, it appears beneficial to specifically address the expectations of GPs regarding the services to be provided by guidelines. This includes making them simple and concise and reducing guideline complexity, and not only applying this to the abstracts (e.g., more focus on visualisations and algorithms) [32]. It should also be ensured that guidelines grant appropriate freedom of action to general practitioners to find practical solutions. Where possible, guidelines should provide the possibility to select between several decision options with regard to diagnostics or treatment as well as they should enable the delegation of tasks among the practice teams to help relieve the burden on physicians and make the implementation more effective [33]. Furthermore, if guidelines are used as standard treatment the status of liability becomes important. Therefore, general practitioners should be certain that following a guideline will not lead to legal problems.

In addition, it should be ensured that the requirements of physicians with a sceptical stance are taken into account during guideline development. Regarding general practitioners’ involvement in the development process, professional associations such as DEGAM already apply a participative approach. Therefore, it may be advisable to improve the communication of the guideline process to general practitioners.

## Supplementary Information

Questionnaire
