# Supplementary material for: What is the significance of guidelines in the primary care setting? Results of an exploratory online survey of general practitioners in Germany
Source: Wien Med Wochenschr. 2021 Jun 8;171(13-14):321–9. doi: 10.1007/s10354-021-00849-3 (PMC8484242; doi:10.1007/s10354-021-00849-3)
Supplement: Supplementary file 1 — Questionnaire [file 10354_2021_849_MOESM1_ESM.docx]

**1. In general terms, do you have more of a positive or more of a negative opinion on guidelines?**

⃝ Very positive ⃝ Somewhat positive ⃝ Somewhat negative ⃝ Very negative ⃝ Difficult to say, no answer

**2. In your opinion, or based on your experience, how useful are guidelines for patient care?**

⃝ Very useful ⃝ Somewhat useful ⃝ Somewhat less useful ⃝ Not very useful ⃝ Not useful ⃝ Difficult to say, no answer

**3. Guidelines are meant to help reduce certain types of problems in the healthcare system. In your opinion, or based on your experience, how good are guidelines at helping achieve the following objectives?**

|  | Very good | Somewhat good | Not very good | Not good at all |
| --- | --- | --- | --- | --- |
| Increased evidence-based approach and application of current medical knowledge | ⃝ | ⃝ | ⃝ | ⃝ |
| Improving care and treatment quality, reducing overprovision, underprovision or incorrect provision of care | ⃝ | ⃝ | ⃝ | ⃝ |
| Ensuring diagnosis and treatment standards for a consistent approach by physicians | ⃝ | ⃝ | ⃝ | ⃝ |
| Improving cooperation between various types of care providers (e.g., GPs and specialists) | ⃝ | ⃝ | ⃝ | ⃝ |
| Reducing health care costs | ⃝ | ⃝ | ⃝ | ⃝ |
| Increasing consideration of the needs of the individual patients | ⃝ | ⃝ | ⃝ | ⃝ |

**4. Which of the following statements do you agree with?**

|  | Fully agree | Somewhat agree | Somewhat disagree | Fully disagree |
| --- | --- | --- | --- | --- |
| “Guidelines increase the efficiency of diagnostic procedures and therapies in medical practice .” | ⃝ | ⃝ | ⃝ | ⃝ |
| “I prefer to rely on my own approach rather than guidelines.” | ⃝ | ⃝ | ⃝ | ⃝ |
| “Guidelines are generally easy and straightforward to implement.” | ⃝ | ⃝ | ⃝ | ⃝ |
| “Guidelines interfere too much in established practice procedures and routines.” | ⃝ | ⃝ | ⃝ | ⃝ |
| “Guidelines enable a more structured approach to diagnosis and therapy.” | ⃝ | ⃝ | ⃝ | ⃝ |
| “Guidelines place too many restrictions on therapy freedom.” | ⃝ | ⃝ | ⃝ | ⃝ |
| “The recommendations for action provided in guidelines often coincide with my personal experience as a physician.” | ⃝ | ⃝ | ⃝ | ⃝ |
| “There is often not enough time in the day-to-day practice setting to implement guidelines.” | ⃝ | ⃝ | ⃝ | ⃝ |

**5. How often do you use or refer to guidelines, for example, for diagnostics, therapy, or patient management?**

⃝ Often ⃝ Occasionally ⃝ Rarely ⃝ Never => Go straight to Question 12 ⃝ Prefer not to answer

**6. More specifically, in which situations do you use or refer to guidelines or when have you done so in the past?**

|  | Often | Occasionally | Rarely | Never |
| --- | --- | --- | --- | --- |
| In suspected cases | ⃝ | ⃝ | ⃝ | ⃝ |
| During the initial diagnosis | ⃝ | ⃝ | ⃝ | ⃝ |
| During therapy or disease management | ⃝ | ⃝ | ⃝ | ⃝ |
| During check-ups or monitoring | ⃝ | ⃝ | ⃝ | ⃝ |
| For screening purposes | ⃝ | ⃝ | ⃝ | ⃝ |
| In emergency situations | ⃝ | ⃝ | ⃝ | ⃝ |
| For specialist diagnoses, i.e., rare diseases | ⃝ | ⃝ | ⃝ | ⃝ |
| For prevention purposes | ⃝ | ⃝ | ⃝ | ⃝ |

**7. Generally in your experience, how has the application of the guidelines you have used so far had an impact on overall care and treatment quality?**

⃝ Very positive ⃝ Somewhat positive ⃝ Somewhat negative ⃝ Very negative

⃝ No effect ⃝ Difficult to say, Prefer not to answer

**8. Which of the following statements correspond to your own experience using guidelines?**

|  | Fully agree | Somewhat agree | Somewhat disagree | Fully disagree |
| --- | --- | --- | --- | --- |
| “I have learnt new things related to diagnosis or therapy by implementing guidelines.” | ⃝ | ⃝ | ⃝ | ⃝ |
| “In general, for pharmaceutical therapy, I follow guideline recommendations” | ⃝ | ⃝ | ⃝ | ⃝ |
| “It is difficult for me to imagine forgoing the use of guidelines in the practice setting.” | ⃝ | ⃝ | ⃝ | ⃝ |
| “By using guidelines, I have been able to improve my diagnostic/therapeutic skills.” | ⃝ | ⃝ | ⃝ | ⃝ |
| “The advantages of guidelines outweigh the disadvantages.” | ⃝ | ⃝ | ⃝ | ⃝ |

**9. How did you originally find out about the guidelines you have implemented?** (Multiple answers possible)

⃝ Specialist journals ⃝ Training courses ⃝ Textbooks/reference books ⃝ Quality circle work

⃝ Internet ⃝ Colleagues ⃝ Degree course, specialisation training

⃝ Info material, brochures from foundations ⃝ Info material, brochures from pharmaceutical companies

⃝ Other, namely: ____________________________________________

⃝ I don’t remember ⃝ Prefer not to answer

**10. How often have you experienced problems or complications at your practice due to the application of guidelines?** (i.e., delays/difficulties in practice processes)

⃝ Often ⃝ Occasionally ⃝ Rarely ⃝ Never ⃝ Difficult to say, Prefer not to answer

**11. In the daily practice setting, how often do you deviate from implemented guidelines, e.g., you are not able to exactly follow recommendations or standards?**

⃝ Often ⃝ Occasionally ⃝ Rarely ⃝ Never ⃝ Difficult to say, Prefer not to answer

**12. There are various guidelines for different symptom domains and clinical pictures. These guidelines (S2 and S3) are provided by the German College of General Practitioners and Family Physicians (DEGAM). Which of the following guidelines are you aware of and which have you implemented?**

|  | Aware of | Aware of and implemented |
| --- | --- | --- |
| Burning sensation when urinating (S3) | ⃝ | ⃝ |
| Fatigue (S3) | ⃝ | ⃝ |
| Family caregivers (S3) | ⃝ | ⃝ |
| Sore throat (S3) | ⃝ | ⃝ |
| Stroke (S3) | ⃝ | ⃝ |
| Acute and chronic coughing (S3) | ⃝ | ⃝ |
| Acute vertigo in the GP practice setting (S3) | ⃝ | ⃝ |
| Chest pain (S3) | ⃝ | ⃝ |
| General practitioner preventive consultation for cardiovascular risk (S3) | ⃝ | ⃝ |
| Multimorbidity (S3) | ⃝ | ⃝ |
| Care of renal insufficiency not requiring dialysis (S3) | ⃝ | ⃝ |
| Ear pain (S2k) | ⃝ | ⃝ |
| Rhinosinusitis (S2k) | ⃝ | ⃝ |
| Elevated TSH values in the GP practice setting (S2k) | ⃝ | ⃝ |
| Application of new platelet aggregation inhibitors (S2e) | ⃝ | ⃝ |
| General practitioner guidelines: Multimedication (S2e) | ⃝ | ⃝ |
| Protecting against overprovision or underprovision of care – Deciding together (S2e) | ⃝ | ⃝ |

**13. In general, do you prefer to implement DEGAM guidelines or is it of no importance to you if they are published by another professional society as long as they have a clear reference to general practitioners?**

⃝ I prefer DEGAM ⃝ It is of no importance ⃝ Difficult to say, Prefer not to answer

**14. Are there specific symptoms or diseases for which you would like to have genuine DEGAM guidelines?**

__________________________________________________________________________________

__________________________________________________________________________________

__________________________________________________________________________________

**15. In your opinion, what must a good set of guidelines have for you to consider implementing it?** **Please indicate how important the following aspects are to you.**

|  | Very important | Somewhat important | Not very important | Not at all important |
| --- | --- | --- | --- | --- |
| The benefits of its recommendations for action must be evidence-based and scientifically valid. | ⃝ | ⃝ | ⃝ | ⃝ |
| It must specify red flags, i.e., particularly important warning signs that indicate clinical pictures in need of further clarification. | ⃝ | ⃝ | ⃝ | ⃝ |
| It should be an S3 guideline (highest level). | ⃝ | ⃝ | ⃝ | ⃝ |
| It should provide easily comprehensible algorithms or diagnostic and therapeutic approaches (i.e., using diagrams). | ⃝ | ⃝ | ⃝ | ⃝ |
| It should be as easy as possible to implement. | ⃝ | ⃝ | ⃝ | ⃝ |
| It should provide clear information on when or for how long a wait-and-see approach is appropriate and when referral is indicated. | ⃝ | ⃝ | ⃝ | ⃝ |
| It should provide concrete ranges for laboratory values (e.g., for blood testing). | ⃝ | ⃝ | ⃝ | ⃝ |
| The recommendations for action should conform to the applicable fee schedule to ensure that physician costs are covered. | ⃝ | ⃝ | ⃝ | ⃝ |
| It must be ensured that the recommendations have a sound legal basis. | ⃝ | ⃝ | ⃝ | ⃝ |
| Guideline-compliant training courses must be available. | ⃝ | ⃝ | ⃝ | ⃝ |
| The guidelines must provide intelligent recommendations for the delegation of tasks for the entire practice team. | ⃝ | ⃝ | ⃝ | ⃝ |
| General practitioners should be involved when developing guidelines or have tested guidelines in a practice setting before publication. | ⃝ | ⃝ | ⃝ | ⃝ |

**16. Below are some recommendations for improvements that could be made to guidelines for general practitioner use. Which of these are particularly important to you? Please choose a maximum of three.**

⃝ Rapid updating of guidelines, faster revision

⃝ A more compact guideline design, kept as short and concise as possible (e.g., more diagrams)

⃝ Involve general practitioners more closely and consistently in the development of guidelines

⃝ More consideration given to non-drug alternatives when developing guidelines

⃝ Accurate information relating to the efficacy of the recommended therapy or therapies

⃝ Inclusion of alternative medicine

⃝ Comprehensive information on the cost of the recommended diagnostics or therapies

⃝ Comparative assessment of various treatment options

⃝ Exact medication dosages

⃝ More thought put into questions regarding quality of life

⃝ A wider availability of guideline-compliant training opportunities to support the effective implementation of guidelines in the practice setting

**17. Imagining that the points you have chosen above are put into practice, under these conditions, would you be more willing than before to implement or refer to guidelines?**

⃝ Yes, much more ⃝ Yes, somewhat more ⃝ No ⃝ Prefer not to answer

*Finally, we need some statistical information from you. As with the rest of the questionnaire, all information is handled confidentially and anonymised.*

**Are you…**

⃝ Male ⃝ Female ⃝ Other

Your **age**: _____

Your practice is located in a **municipality/town with…**

⃝ More than 100,000 ⃝ 20,000 to 100,000 ⃝ 5,000 to 20,000 ⃝ Fewer than 5,000 inhabitants

**Which model best describes your practice**?

⃝ Single-partner practice (You are the only physician) ⃝ Single-partner practice with contracted physicians

⃝ Joint practice ⃝ Medical care centre ⃝ Other

**How many patients does your practice treat every quarter**?

⃝ 500 to 750 ⃝ 751 to 1,000 ⃝ 1,001 to 1,500 ⃝ 1,501 to 2,000 ⃝ More than 2,000

**Thank you for participating in this questionnaire!**
